# Functional promoter testing using a modified lentiviral transfer vector

**Published:** 2007-05-17

**Authors:** Scott F. Geller, Phillip S. Ge, Meike Visel, Kenneth P. Greenberg, John G. Flannery

**Affiliations:** 1 Helen Wills Neuroscience Institute, University of California, Berkeley, CA; 2 Department of Molecular and Cell Biology, University of California, Berkeley, CA; 3 Department of Vision Science, University of California, Berkeley, CA

## Abstract

**Purpose:**

The importance of retinal glial cells in the maintenance of retinal health and in retinal degenerations has not been fully explored. Several groups have suggested that secretion of neurotrophic proteins from the retina's primary glial cell type, the Müller cell, holds promise for treating retinal degenerations. Tight regulation of transgene expression in Müller cells is likely to be critical to the efficacy of long-term neuroprotective therapies, due to the genetic heterogeneity and progressive nature of retinal disease. To this end, we developed a modified lentiviral (LV) transfer vector (pFTMGW) to accelerate the testing and evaluation of novel transcriptional regulatory elements. This vector facilitates identification and characterization of regulatory elements in terms of size, cell specificity and ability to control transgene expression levels.

**Methods:**

A synthetic multiple cloning site (MCS) which can accept up to five directionally cloned DNA regulatory elements was inserted immediately upstream of an enhanced green fluorescent protein (eGFP) reporter. A cytomegalovirus (CMV) promoter, required for tat-independent viral packaging, is located around 2 kb upstream of the eGFP reporter and is capable of directing transgene expression. A synthetic transcription blocker (TB) was inserted to insulate the MCS/eGFP from the CMV promoter. We evaluated eGFP expression from pFTMGW and control constructs using flow cytometry and quantitative reverse transcriptase polymerase chain reaction (RT-PCR). We also tested and compared the activity and cell specificity of a computationally identified promoter fragment from the rat vimentin gene (Vim409) in transfection and lentiviral infection experiments using fluorescence microscopy.

**Results:**

Transfection data, quantitative RT-PCR, and flow cytometry show that around 85% of expression from the CMV promoter was blocked by the TB element, allowing direct evaluation of expression from the Vim409 candidate promoter cloned into the MCS. Lentiviruses generated from this construct containing the Vim409 promoter (without the TB element) drove robust eGFP expression in Müller cells in vitro and in vivo.

**Conclusions:**

The TB element efficiently prevented eGFP expression by the upstream CMV promoter and the novel MCS facilitated testing of an evolutionarily conserved regulatory element. Additional sites allow for combinatorial testing of additional promoter, enhancer, and/or repressor elements in various configurations. This modified LV transfer vector is an effective tool for expediting functional analysis of gene regulatory elements in Müller glia, and should prove useful for promoter analyses in other cell types and tissues.

## Introduction

Re-engineered viruses or "vectors" are a widely used tool for nucleic acid delivery, transgene expression, and gene therapy [1]. Adeno-Associated Virus (AAV) is a commonly used gene therapy vector [2], with several positive attributes for gene delivery. Unfortunately the physical size of the AAV capsid (25 nm) appears to limit the length of the transgene "payload" to about 4.7 kb, which includes the required inverted terminal repeats (143 bp each), the cDNA "cargo," and any regulatory elements necessary for cell-specific targeting and expression [3,4]. AAV serotype 2 (AAV2) is the most commonly used vector for gene transfer to the eye. This single stranded DNA vector typically exhibits a delay (generally 2-3 weeks) in the onset of transgene expression [5] in vivo. This lag in expression is thought to be due to the time required for trafficking of the virus to the nucleus, capsid uncoating, and subsequent stabilization by single- to double-stranded conversion of the viral genome [6]. For purposes of promoter optimization, this significantly increases the time required to fully evaluate regulatory elements and/or the effects of therapeutic molecules, particularly in the context of developmental studies and/or the evaluation of therapeutics for rapidly progressing diseases. Recently, double-stranded AAV vectors have been developed [7] that efficiently express their transgene within days, although the inclusion of the second strand further reduces the carrying capacity to <2.5 kb [8].

In contrast, lentiviral (LV) vector capsids have a larger physical size (about 100 nm) and are capable of packaging promoter/transgene sequences over twice that of AAV [9]. This property is invaluable for transfer of large promoter constructs or transgene coding sequences which cannot be accommodated within AAV vectors [2,10,11]. Furthermore, concentration and purification of LV vectors may be accomplished by ultracentrifugation alone, whereas AAV vectors require the use of column chromatography to generate pure high titer preparations.

Another important distinction is that in contrast to AAV, lentiviruses are enveloped RNA viruses whose genome is reverse-transcribed into double-stranded DNA by the viral reverse transcriptase soon after entering the target cell. The double-stranded DNA genome is then readily integrated into the host genome by the included viral integrase. As a result, LV vector-mediated transgene expression in non-dividing (and dividing) cells begins shortly after infection (about 24-48 h), greatly improving the speed in which experimental promoters and/or therapies may be evaluated in vivo. Furthermore, their envelope can be readily modified by insertion of diverse array of glycoproteins (e.g., xenobiotic or orthologous), allowing for manipulation of viral tropism and cellular targeting [12].

Developing improved viral vectors possessing precise regulatory control over transgene expression is a major focus of gene therapy research. Inclusion of cloned promoter elements has been the primary approach used to direct and control transgene expression. However, despite recent advances in bioinformatics [13-17] and whole genome sequence information [18,19], predicting how the DNA sequence and genomic context combine to control and regulate mRNA expression remains an inexact science. Currently, there is no reliable method to predict the manner in which promoter and enhancer sub-sequences influence and control levels of expression, impart positive or negative modulation, or determine cell-type specific gene expression patterns in mammalian tissues [20].

As additional whole genome sequences become available, and as computational methodologies continue to improve, the process for determining which sequences are both functional and relevant for cell- and context-specific gene expression will become less empirical. Nevertheless, regulatory elements will continue to require in vivo evaluation before therapeutic use in humans can be considered, particularly when expressing transgenes in diverse multicellular tissues. Until transgenes can be integrated in a site-specific manner (i.e., to the endogenous genomic locus control region), cloned fragments of gene promoters will continue to be used to direct expression. Though large promoter elements from individual genes are often sufficient for general laboratory procedures, it is highly desirable to improve therapeutic control over transgene expression, and to reduce the size of regulatory elements used in experimental vectors. Increasing the speed at which small, compound promoters can be evaluated in vitro and in vivo will help to identify and characterize promoters for use in basic biology and molecular therapies. Here we detail modifications made to a LV transfer vector [21] that allows for efficient evaluation and testing of potentially complex promoter elements. The vector reduces the need to shuttle promising regulatory elements between plasmids, permits simultaneous testing of multiple elements for combinatorial effects on expression of a cDNA payload, and facilitates the identification of smaller promoter elements, enabling a broader use of viruses with more limited cloning capacities.

## Methods

### Creating the pFTMGW plasmid

**Figure 1:**
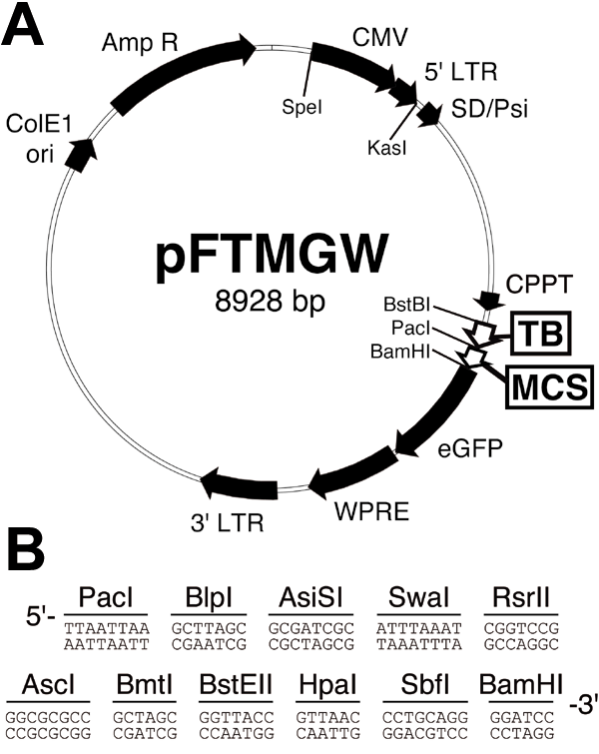
Plasmid map of the pFTMGW vector. **A**: Two primary modifications were made to the parent vector, pFUGW; both are surrounded by rectangles. First, a novel multiple cloning site (MCS) replaced the human ubiquitin-C promoter. Second, a transcription blocker (TB) was cloned immediately upstream of the MCS to prevent aberrant expression from the upstream CMV promoter. **B**: The sequence (5'-3') of restriction enzyme sites included in the new MCS. ColE1 ori (bacterial origin of replication) and AmpR (ampicillin resistance gene) are used for plasmid replication. The enhanced green fluorescent protein (eGFP) is the reporter molecule. The CMV (cytomegalovirus promoter), LTRs (long terminal repeats), SD/Psi (splice donor and viral packaging sequences), CPPT (central polypurine tract), and WPRE (Woodchuck hepatitis virus post-transcriptional regulatory element) are virus-related elements.

A synthetic multiple cloning site (MCS, Figure 1B) containing 11 unique restriction sites replaced the ubiquitin-C promoter (*Pac*I to *Bam*HI) in pFUGW (a gift of Dr. David Baltimore, California Institute of Technology), thereby creating pFMGW. To reduce non-specific transgene expression driven by the hybrid CMV/LTR, a 154 bp synthetic transcription blocker [22-25] (TB; also see: pCI-neo vector (Promega, Madison, WI) and pSEAP2 vector (Clontech Laboratories, Inc., Mountain View, CA)) was PCR amplified (Forward: 5'-TTC GAA AAT AAA ATA TCT TTA TTT TCA TTA CAT CTG TGT GTT GGT T-3', *BstB*I site in red color; Reverse: 5'-TTA ATT AAA GAG AAA TGT TCT GGC ACC-3', PacI site in red color) from an existing plasmid in our laboratory (pAAV-6P1-TB; a gift from Dr. Sebastian Kügler, University of Göttingen Medical School, Germany [26]). The TB element was cloned into the PacI site immediately upstream of the MCS, preserving a PacI site between the TB and MCS. The resulting plasmid was named pFTMGW (Figure 1A). The sequence of the pFTMGW plasmid has been submitted to GenBank, accession number: EF177827.

### Cloning the Vim409 promoter fragment

We cloned a 409 bp fragment (Vim409; rat genome build rn4, chr17:87846950-87847358; see rat rn4) of the vimentin gene promoter/5' UTR from Sprague-Dawley (SD) genomic DNA into the pFTMGW vector. The Vim409 fragment lies immediately upstream of the vimentin coding sequence (not including the bases CATG at the translational start site, red color) The primers for cloning Vim409 were supplemented with HpaI (forward, in red color) and BamHI (reverse, in red color) restriction sites for directional cloning into pFTMGW: Forward, 5'-GTT AAC CGC GAT CCC TTC TTT CTC AGC AC-3'; Reverse, 5'-GGA TCC GCT TCG AAG GAC GAG GTG GCC-3'. The sequence was computationally identified using freely available software at DCODE [27], mandating a minimum of 75% homology between rat and human sequences over a 250 bp "window." The resultant Vim409 fragment shares 75.79% (310/409) homology with the human vimentin promoter, including 6 small gaps.

### Cell culture

Rat Müller cells (a gift from Dr. Rong Wen, University of Pennsylvania) were cultured under standard culture conditions (37 °C, 5% CO_2_, 95% air) in DMEM (Cambrex Corp., East Rutherford, NJ), 10% fetal bovine serum (HyClone, Logan, UT), 4 mM L-glutamine (Invitrogen Corp., Carlsbad, CA) and 1X antibiotics (Penicillin (100 U/ml) and Streptomycin (100 μg/ml; Invitrogen)) [28,29]. T24 cells (ATCC HTB-4) were cultured and propagated similarly to the Müller cells, except that we used 2 mM L-glutamine, and supplemented with 100 nM non-essential amino acids (Invitrogen), and 1 mM sodium pyruvate (Invitrogen).

### Cell transfection and infection

**Figure 2:**
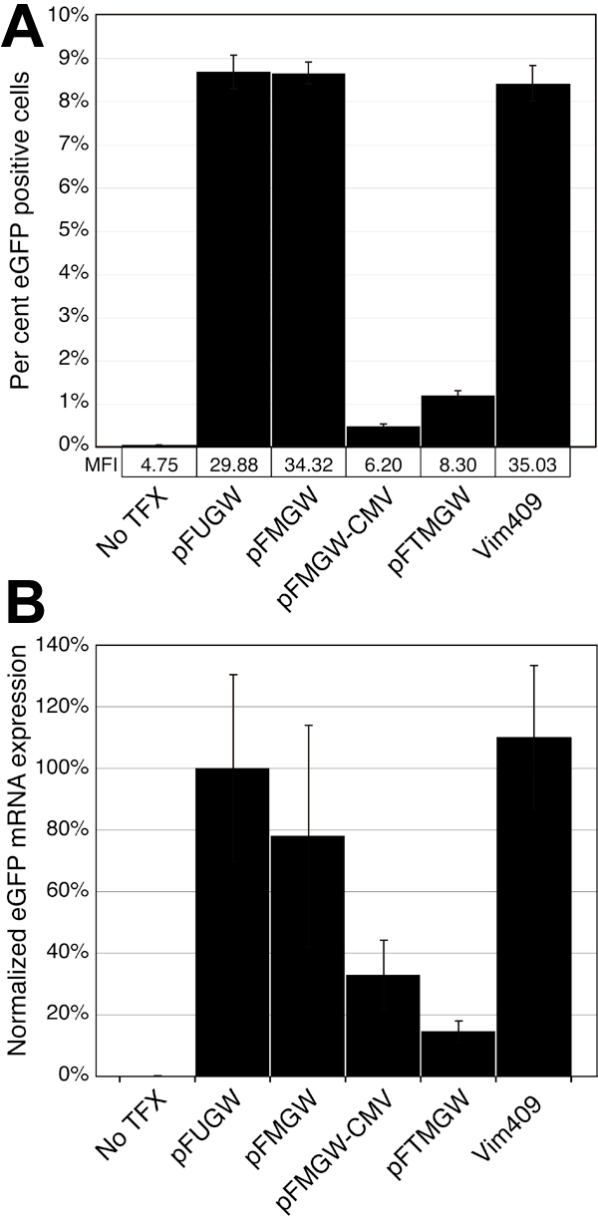
Quantitative analysis of eGFP expression in transfected cells. **A**: Flow cytometry analysis of eGFP expressing Müller cells. Nearly confluent cells in 6-well plates were un-transfected (No TFX), transfected with 2 μg of control vectors (pFUGW, pFMGW, pFMGW-CMV, pFTMGW), or transfected with 2 μg of a 409 bp fragment of the rat vimentin promoter cloned into pFTMGW (Vim409). Greater than 10^5^ cells were analyzed in each of six replicate samples for each condition. Gating was set such that positive cell counts in the No TFX samples were less than 0.07% of all cells. **B**: Quantitative RT-PCR analysis of eGFP expression. Total RNA was isolated from cultured Müller cells transfected with the same set of plasmids as in **A**, and reverse transcribed. eGFP and β-actin transcript expression were measured using Taqman fluorescent probes. Threshold cycle numbers were first internally normalized (within each individual reaction) to β-actin, and secondarily normalized to pFUGW eGFP expression levels (set to 100%). Samples were analyzed in triplicate. pFUGW is the parent plasmid. pFMGW is pFUGW with the MCS in place of the ubiquitin-C promoter. pFMGW-CMV is the pFMGW plasmid lacking the CMV promoter. pFTMGW is pFMGW with the TB cloned immediately upstream of the MCS. Vim409 is the 409 bp fragment of the rat vimentin promoter/5' UTR cloned into pFTMGW. Error bars represent 1 SD for panels **A** and **B**.

One sterilized square cover slip (22 mm number 1, Fisher Scientific, Hampton, NH) was placed into each well of 6-well plates, and coated with poly-L-lysine (diluted 1:10 with PBS; Sigma-Aldrich, Milwaukee, WI) for 10 min. One day before transfection, rat Müller or T24 cells were seeded at 0.8-1x10^6^ cells per well in media without antibiotics. When about 90% confluent, the cells were transfected with 2 μg of plasmid DNA (see below; Figure 2) complexed with Lipofectamine 2000 reagent according to the manufacturer's protocol (Invitrogen). For infection with LV, cells were seeded at 1x10^5^ with media (as above), and 1 μl virus was added the following day.

Twenty-four hours following either transfection or infection, the culture medium was removed, the cells were rinsed 3 times for 10 min in PBS (pH 7.4), and then fixed with 10% neutral buffered formalin (Ted Pella, Inc., Redding, CA) at room temperature for 15 min. Cells were also processed at 48 and 72 h post transfection; no difference was observed between the samples at different time points (data not shown). Cells (grown on cover slips) were rinsed 3 times with PBS and sealed onto microscope slides with Vectashield hard set mounting medium containing DAPI (Vector Laboratories, Inc., Burlingame, CA) using nail polish. Cells were digitally imaged on a Zeiss Axiophot fluorescence microscope with an MRc5 camera (Carl Zeiss, Oberkochen, Germany).

### Flow cytometry

Rat Müller cells grown directly on 6-well plates were either untreated (No TFX) or transfected with 2 μg pFUGW, pFMGW, pFMGW-CMV, pFTMGW, or Vim409 (see above; also see Figure 2). All plasmids utilized eGFP as the reporter molecule, and samples were prepared in sextuplicate. After 24 h, Müller cells were washed with PBS and treated with 0.1 ml per well of 0.25% trypsin (Invitrogen). Culture media was added (0.9 ml) and cells were counted on a EPICS XL-MCL flow cytometer (Beckman-Coulter, Inc., Fullerton, CA), using 488 nm excitation. At least 10^5^ cells were analyzed, and mean fluorescence intensity (MFI) and percent eGFP positive cells were calculated for each sample under identical gating conditions.

### Quantitative reverse transcriptase polymerase chain reaction

For quantitative RT-PCR analysis, total RNA was isolated 24 h after transfection using RNeasy columns (Qiagen Corp., Valencia, CA) followed by DNAse I treatment (Sigma; to eliminate residual DNA). One μg of total RNA was reverse transcribed (RT) for 2 h at 37 °C for each sample (three per condition) using random hexamers (Invitrogen) and MMLV-RT (Promega). One μl (50 ng) was used in each 20 μl PCR reaction, performed in technical replicate. The primers were as follows: eGFP forward, 5'-AGC AGC ACG ACT TCT TC-3'; eGFP reverse, 5'-TCG TCC TTG AAG AAG ATG-3'; Actin forward, 5'-ACC AAC TGG GAC GAC ATG GAG AA-3'; Actin reverse, 5'-CAT GGC TGG GGT GTT GAA GGT-3'. We used an eGFP probe (5'-FAM-AGT CCG CCA TGC CCG AAG GCT-BHQ-3') and a beta-actin probe for internal normalization of each sample (5'-CAL560-CTG GCA CCA CAC CTT CTA CAA TGA GC-BHQ-3'); both of which were designed (online assays) and ordered from Biosearch Technologies (Novato, CA). Amplification was performed on an Mx3000P real-time thermal cycler (Stratagene Corp., La Jolla, CA). Samples were denatured for 10 min at 95 °C and then cycled 40 times (30 s at 95 °C, 2 min at 53 °C). We used 300 nM final concentration of primers and probes, 0.1 U per reaction Platinum Taq Polymerase (Invitrogen), and 2.5 mM MgCl_2_. Data were analyzed using Stratagene MxPro software version 3.0, and amplification-based threshold cycle (Ct) values were exported to Excel (Microsoft Corp., Redmond, WA). Ct values for eGFP were normalized to beta-actin to control for variability in amounts of input cDNA, and secondarily normalized to pFUGW expression levels (100%, Figure 2D).

### Lentivirus production

Lentivirus was packaged by transfecting 293T cells with Lipofectamine 2000 (in IMDM, 10% FBS, and 2 mM L-glutamine) using a 4-plasmid system consisting of the Vim409 transfer vector, pMDLg/pRRE, pRSV-REV, and pMD.G (VSV-G [30,31]) as previously described [32]. Briefly, the cells were plated the previous day in three T-175 flasks (coated with poly-L-lysine) at a density of 1.5x10^7^ cells/plate. Twelve hours after transfection, media with 1X antibiotics (Penicillin (100 U/ml) and Streptomycin (100 μg/ml; Invitrogen)) replaced the media containing the transfection reagents. Supernatants (containing the viral particles) were harvested 24 and 48 h after the first media change, and filtered through a 0.45 μm pore PVDF Durapore filter (Millipore Corp., Billerica, MA). The filtrates were centrifuged in 4 ultracentrifuge tubes (38.5 ml; number 344058; Beckman-Coulter) underlaid with 4 ml of sucrose (20%) using a SW-28 rotor spinning at 103,864xg for 2 h at 4 °C. The pellets were resuspended in 800 μl cold PBS. After 30 min incubation on ice, the samples were pooled and centrifuged in a SW-41Ti rotor for 1.5 h at 105,462xg at 4 °C. The pellet was carefully resuspended in 120 μl cold PBS. After incubation on ice for 2 h the virus was divided into 20 μl aliquots, and used immediately or stored at -80 °C.

### Viral titer determination

RNA samples from DNAse-treated Vim409 virus preparations (5.0, 1.0, 0.5, 0.1, and 0.05 μl) were purified using RNeasy columns (Qiagen Corp., Valencia, CA). The RNA was reverse transcribed as above, and 1 μl served as template for qPCR analysis using a dual-labeled probe to eGFP. The standard curve was determined using plasmid DNA and the viral titer was calculated to be about 2x10^13^ vector genomes (VG) per ml.

For functional titer determination, 293T cells (2x10^5^ cells per well in a six well plate) were infected with 1.0, 0.5, and 0.25 μl Vim409 virus as described previously [33]. After seven days DNA from 1x10^6^ cells was isolated using a Puregene Genomic DNA Purification Kit (Gentra Systems, Minneapolis, MN), and qPCR analysis indicated a titer of about 5x10^11^ transducing units (TU) per ml.

### Subretinal injection

All animals were treated in compliance with the ARVO (Association for Research in Vision and Ophthalmology) Statement for the Use of Animals in Ophthalmic and Visual Research, and all procedures were in accordance with protocols approved by the Animal Care and Use Committee, University of California, Berkeley. Protocols for subretinal injections have been thoroughly noted by ourselves and others [32-37]. Briefly, after deep anesthesia, and after a pilot hole was created at pars plana of the eye, a Hamilton syringe fitted with a 33-gauge blunt needle containing 3 μl of virus was inserted into the eye, and the virus was injected under the retina. The needle was removed slowly, and the animals recovered on a warming blanket.

### Fundus and confocal microscopy

Prior to sacrifice, eyes were examined using a RetCam fundus camera (Clarity Medical Systems, Inc., Pleasanton, CA). Animals were deeply anesthetized by intraperitoneal injection with a mixture of xylazine (13 mg/kg) and ketamine (87 mg/kg). Corneas were directly anesthetized with drops of proparicane (0.5%), pupils were dilated with phenylephrine (2.5%) and atropine (1%) drops, and fluorescent in vivo fundus images were collected. Animals were euthanized by CO_2_ overdose followed by cervical dislocation, and eyes were enucleated and fixed in 10% neutral buffered formalin (Ted Pella). The cornea and lens were removed and the posterior pole was returned to fixative for 2 h, rotating at 4 °C. After PBS rinses (3x10 min), the eyecup was embedded in Tissue Freezing Medium (TBS, Durham, NC). Eyes were cryosectioned at 30 μm, and sections were collected on ProbeOn Plus microscope slides (Fisher Scientific). Sections were mounted under cover slips with Vectashield mounting medium containing DAPI (Vector Laboratories). eGFP positive retinal sections were analyzed on an Axioplan 510 Meta confocal microscope (Carl Zeiss).

## Results

### Quantitative analysis

We generated a lentiviral vector backbone that incorporated a large MCS in place of the ubiquitin-C promoter (pFMGW). By replacing the cloned ubiquitin-C promoter with the MCS, we anticipated a complete loss of eGFP expression in transfection experiments. However, using both flow cytometry (Figure 2A) and quantitative RT-PCR (Figure 2B), we observed that pFMGW continued to express high levels of eGFP in cultured cells (Figure 2A,B). Comparing pFUGW with pFMGW, flow cytometry analysis indicated little change in eGFP protein expression (Figure 2A), and quantitative RT-PCR indicated a modest (about 20%) decrease in mRNA transcript levels (Figure 2B). These results suggested that the upstream CMV promoter was strongly influencing eGFP gene expression. Excising the CMV promoter sequence (pFMGW-CMV) dramatically reduced eGFP expression (Figure 2A,B), indicating that despite a distance of >2 kb upstream, the external CMV promoter was capable of directing high levels of eGFP transgene expression.

As an alternative to removing the CMV/LTR from the backbone vector, a sequence that is necessary for producing high titer viruses in the packaging cell line, we tested the effectiveness of a synthetic TB element inserted immediately upstream of the MCS (Figure 1). The TB element has been previously characterized as containing a synthetic poly-A (SPA) sequence (AATAAA sequence and a GT/T-rich sequence with the correct spacing of 22-23 nucleotides between them) [22], and a C2 transcriptional pause site [23], which slows the processivity of the RNA polymerase complex. Quantitative assessment of TB function by both flow cytometry (Figure 2A) and quantitative RT-PCR (Figure 2B) indicated a reduction in eGFP expression by approximately 85%. A second TB element arranged in tandem did not enhance the blocking effect (data not shown). Moreover, the mean fluorescence intensity (MFI) in the flow cytometry samples (Figure 2) indicated a reduction in the amount of eGFP protein produced in the small population of transfected cells that circumvented the transcriptional block. Cells transfected with pFMGW had a MFI of 34.32, while inclusion of the TB element in pFTMGW reduced the MFI to 8.3.

To validate the suitability of our modified pFTMGW vector for promoter screening in vitro, we measured eGFP expression from a proximal promoter fragment of a glial specific gene cloned into the MCS of pFTMGW. Using comparative genome analysis tools at DCODE [27], we selected and PCR-cloned a promoter fragment (Vim409) from the rat vimentin gene. Transfecting the Vim409 construct resulted in robust eGFP expression in cultured Müller cells. By flow cytometry analysis (Figure 2A), the Vim409 plasmid drove eGFP expression as well as the ubiquitin-C promoter in the parent vector (pFUGW). Similarly, by quantitative RT-PCR analysis, transfection with the Vim409 promoter resulted in equal or slightly higher levels of expression when compared to pFUGW (Figure 2B). These data suggest that the pFTMGW vector is useful for assessing gene expression from promoter sequence(s) cloned into the MCS, without significant "non-specific" expression from upstream *cis*-acting elements.

### Qualitative analysis

**Figure 3:**
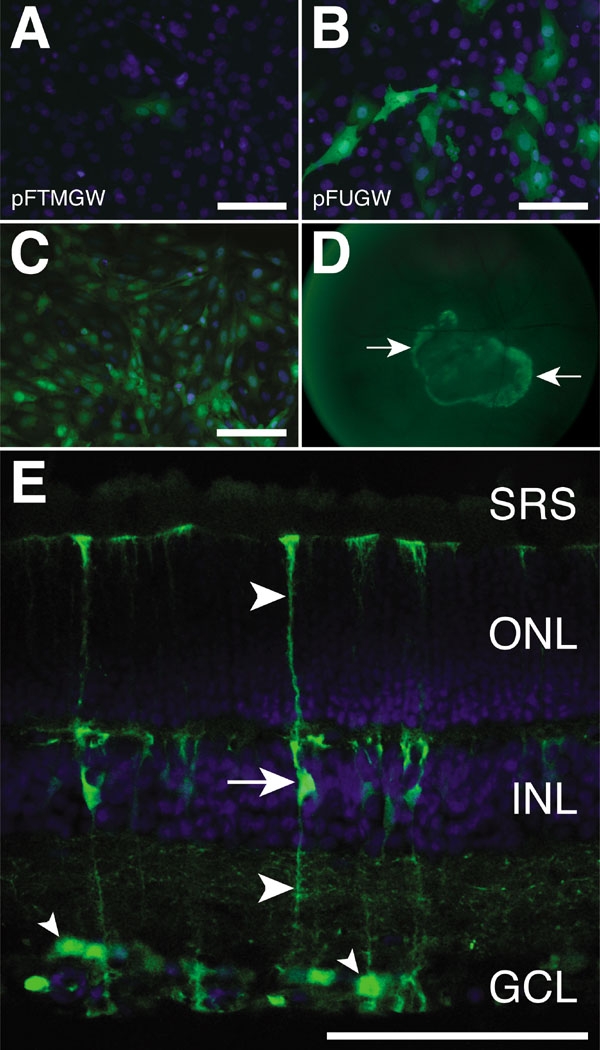
Microscopic evaluation of eGFP expression in Müller cells. **A**: Müller cells transfected with 2 μg of the modified LV transfer vector (pFTMGW) for 24 h. **B**: Müller cells transfected with 2 μg of the parent pFUGW plasmid for 24 h. Note the reduction in eGFP expression when the TB and MCS elements are introduced. **C**: Cultured Müller cells infected with the Vim409 virus expressing eGFP. **D**: An adolescent SD rat injected subretinally with 3 μl of the Vim409 virus. Shown here is an in vivo fluorescent fundus photograph of an eye, 5 days after injection. Arrows denote the area of viral infection and consequent eGFP expression. **E**: The eye shown in **D** was removed and processed for confocal microscopy. Shown here is a confocal image of a retinal cross-section, 5 days following infection with the Vim409 virus. The arrow indicates eGFP expression in Müller cell bodies, and large arrowheads identify the characteristic Müller cell processes that span the thickness of the neural retina. The small arrowheads indicate astrocytes in the ganglion cell layer (GCL), the other predominant glial cell type in the retina, which also express vimentin. SRS, subretinal space; ONL represents outer nuclear layer; INL represents inner nuclear layer. Scale bars in **A**, **B**, **C**, and **E** represent 100 μm.

Microscopic evaluation of Müller cells transfected with pFTMGW (Figure 3A) also demonstrated significantly reduced numbers of eGFP positive cells when compared with pFUGW (Figure 3B). Though this was an important improvement for transfection studies, one of our primary goals was to create a vector that can be used to quickly screen promoters in vivo as well as in vitro. To generate high titer viral vectors, the TB was removed before packaging. To accomplish this we engineered BstBI and PacI sites to flank the TB, allowing for efficient removal, filling-in, re-ligation, and transformation: all of which can be completed in less than a day. We removed the TB from the Vim409 construct and generated infectious Vim409 LV particles (see methods). The Vim409 virus infected and expressed eGFP in Müller cells, both in vitro (Figure 3C) and in vivo (Figure 3D,E). Cultured Müller cells expressed eGFP within 24 h of infection with 1 μl of the Vim409 LV vector (Figure 3C). Similarly, 5 days after a 3 μl subretinal injection of Vim409 LV vector into rat eyes, eGFP expression was apparent in vivo in a fluorescent fundus photograph (Figure 3D). In retinas examined by conventional fluorescence and confocal microscopy, eGFP expression was apparent in the radial Müller glia and in some presumed astrocytes, cell types that normally express vimentin (Figure 3E).

**Figure 4:**
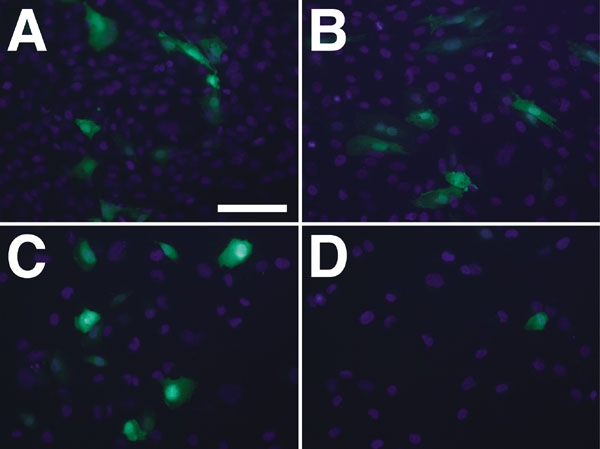
Failure of Vim409 to drive eGFP expression in cells that lack endogenous vimentin expression. **A**: Müller cells transfected with 2 μg of the Vim409 plasmid construct for 24 h. The construct included the upstream hybrid CMV/LTR and the TB element. **B**: Müller cells transfected with 2 μg of the Vim409 plasmid after removing the CMV/LTR. Expression of eGFP was indistinguishable between **A** and **B**. **C**: Cultured T24 cells transfected with 2 μg of the Vim409 plasmid construct for 24 h. **D**: Cultured T24 cells transfected with 2 μg of the Vim409 plasmid after removing the CMV/LTR. Note the near complete loss of expression when the CMV/LTR is removed, validating the specificity of the Vim409 promoter for directing expression in vimentin-positive cells, and indicating that expression in **C** is due to the CMV/LTR promoter driving expression through the TB in a subset of cells. Scale bar in **A** represents 100 μm.

The promoter specificity of the Vim409 construct was validated by transfecting Müller and T24 cells with several plasmid constructs (2 are shown; Figure 4). T24 cells are known to lack vimentin [38] and therefore serve to control for non-specific expression by sequences outside of the MCS-cloned Vim409 promoter. In Figure 4 we show Müller cells (Figure 4A,B) and T24 cells (Figure 4C,D) transfected with Vim409 (Figure 4A,C) and Vim409-CMV (Figure 4B,D). We found that both constructs drove high levels of eGFP expression in vimentin-positive Müller cells with no appreciable difference. These data indicate that Vim409 directs transgene expression in Müller cells as expected, irrespective of the upstream CMV promoter "leaking" through the TB element. In direct contrast, in T24 cells, the Vim409-CMV construct (Figure 4D) fails to drive eGFP expression, while the Vim409 construct continues to show some CMV-driven expression (Figure 4C). These data suggest that the Vim409 promoter is specific for vimentin expressing cells, and that gene-specific promoters can be effectively evaluated using the pFTMGW plasmid backbone vector.

## Discussion

We have developed a vector for rapidly assessing the effectiveness of individual or compound promoter elements to direct gene expression in vitro and in vivo. In the current paper we describe modifications made to a LV transfer vector, and how the modified vector can be used to test cloned promoter elements; in this case, we evaluated the ability of a computationally defined fragment of the vimentin promoter to drive eGFP expression in retinal Müller cells. Recently, we demonstrated that LV vectors containing "full length" vimentin, GFAP, and CD44 promoters can efficiently infect and direct robust eGFP expression in Müller glia [33]. Furthermore, numerous investigators have studied the ability of lentivirus, pseudotyped with diverse envelope glycoproteins and containing various promoters, to infect mammalian retinas [32-36]. These data suggest that the expression of packaged transgenes depends on the combination of pseudotype and promoter elements. Infection of RPE, photoreceptors, and inner retinal cells (including Müller cells) has been demonstrated under such conditions. Several investigators have suggested that Müller glia may be utilized as a cellular conduit for delivering neuroprotective agents and treating retinal disease [39-43]. A necessary concern of any viral-based therapy is how to properly regulate expression of the delivered transgene(s). This vector will enhance our ability to accurately examine unique combinations of regulatory elements and how they influence transgene expression. We are using this vector to better characterize promoters for controlling the expression of transgenes in the context of lentiviral gene therapy. Importantly, however, the vector described here should facilitate promoter analysis in a variety of experimental and therapeutic applications.

The pFUGW plasmid was originally modified [37] by removal of the viral enhancer and promoter elements, and addition of a hybrid 5' LTR generated by replacing the LTR U3 sequence with a CMV promoter. These modifications allow for Tat-independent transcription with no measurable effect on viral titers, and eliminate the *cis*-acting promoter influence in vivo. The original vector was also designed to be self-inactivating, by deleting 133 bp of the U3 region of the 3' LTR, to prevent mobilization of the virus after integration into host cells [37]. We further modified the pFUGW LV transfer vector [21] to expedite the discovery and characterization of shorter regulatory elements capable of directing gene expression in retinal glia. First, we wanted to equip the plasmid with an ample multiple cloning site upstream of eGFP. The MCS enables the ability to test unique combinations of DNA sequences for their ability to regulate transgene expression. For example, up to 5 sub-sequences from the same promoter can be tandemly cloned, arranged, and tested. In this manner, repeated elements or sequences not predicted to be substrates for transcription factor binding/regulation could be eliminated from testing and analysis, if desired. Moreover, hybrid promoters could be designed to include sequences from regulatory regions of different genes. The hope is that hybrid (polygenic) promoters may exhibit unique regulatory characteristics that can be exploited for improving our understanding of gene expression, and possibly for use as regulatory elements in experimental therapies. The resulting vector (pFTMGW) has broad applicability in both in vitro and in vivo assays, and reduces the time and effort required for shuttling, sequencing, screening, and subcloning DNA regulatory elements into different plasmid constructs. The pFTMGW vector can be used to rapidly and efficiently test the functional activity of cloned regulatory elements in both transfection- and infection-based assays. This vector should facilitate the discovery of regulatory elements for retinal gene expression, and will likely be useful for studying promoter/enhancer activity in the brain and other tissues.

We found that the CMV promoter (located over 2 kb upstream of the eGFP) was capable of driving high levels of "non-specific" transgene expression. We felt it was important to prevent undesirable eGFP expression by the external CMV promoter in the plasmid backbone, which is required for Tat-independent virus production [37] in vitro. We tested and determined that a TB element is effective at mitigating the majority (about 85%) of transgene expression from the CMV promoter (Figure 2). Using the Vim409 promoter, we observed that an MCS-cloned promoter directs reporter gene expression in transfected cells at significantly higher levels than control constructs (e.g., pFTMGW). However, it should be noted that because about 15% of gene expression leaks through the TB (in Müller cells), likely due to the robustness of the CMV promoter, it may be worthwhile to excise the CMV/LTR from experimental constructs before in vitro testing. This may be particularly true if the sequence of interest is expected to direct low levels of gene expression or when quantitative measurements are required. It is our experience, nonetheless, that active promoters are readily identified (over and above the CMV-driven expression) during microscopic screening of transfected experimental promoter constructs.

A consideration in the design of the pFTMGW vector was facilitating efficient removal of the TB element to allow transcription of the packaged viral genome (between the two LTRs). TB removal is necessary because the packaged lentiviral RNA genome is produced by Pol II-mediated transcription from the hybrid CMV/LTR [37]. Preventing transcription of the full-length genome by prematurely stopping transcription (by the TB) would block generation of whole RNA transcripts required for packaging into capsids. A restriction digestion reaction to remove the TB element followed by ligation and subsequent plasmid isolation is all that is required for any pFTMGW-based plasmid to become viable for generating infectious LV particles. To validate the ability of these constructs to be used for virus production and subsequent infection assays (Figure 3), we packaged active LV using the Vim409 plasmid after removal of the TB by BstBI and PacI restriction endonuclease digestion (see Figure 1). As shown in Figure 3, the Vim409 virus efficiently infected and expressed eGFP in vimentin-positive cells. Circumventing the need to re-clone fragments into different plasmid backbones should expedite the functional evaluation of experimental promoters.

A common issue in molecular cloning is the presence of a "required" restriction site within a desired DNA fragment; generally precluding a simple cloning procedure, and often necessitating multiple workaround steps. Having 11 unique restriction sites in the MCS (Figure 1B) assures that virtually any small (<1000 bp) DNA fragment can be directionally cloned into at least one pair of sites with relative ease. Furthermore, having numerous sites also allows for tandem, inverted, and combinatorial cloning of regulatory elements into a single "promoter construct," and thus offers the potential to analyze more complex promoter designs.

We demonstrate that a modified LV vector backbone plasmid can be useful for expediting the identification of functional promoter elements. Because LV vector-mediated gene expression is rapid, and tropism can be manipulated by changing the envelope protein expressed in the cell line used for viral packaging [12], we believe that it can be effectively utilized to identify functional, cell-specific promoters in diverse experimental paradigms. We show here that a single vector, pFTMGW, can be used for transfection, flow cytometry, quantitative RT-PCR, and viral infection experiments. Use of this vector should increase throughput and discovery of regulatory elements needed to drive gene expression in a variety of cell types and experimental systems.
